# Hepatic cyst infection complicated by a hepatobronchial fistula diagnosed with unique features

**DOI:** 10.1093/omcr/omab105

**Published:** 2021-10-26

**Authors:** Hatsuo Isogai, Masashi Inoue, Masanao Miura

**Affiliations:** Department of Emergency and Critical Care Medicine, Kariya Toyota General Hospital, Kariya city, Aichi, Japan

## Abstract

A hepatobronchial fistula (HBF) is a rare condition, defined as an abnormal connection of the respiratory system with the liver parenchyma. Although imaging may be helpful for diagnosis, fistulae are often difficult to identify. An 81-year-old woman presented with mild fever and right upper quadrant abdominal pain. Computed tomography (CT) showed bilateral pneumonia and hepatic cyst infection with air-fluid levels. After mechanical ventilation, abdominal CT showed increased air in the hepatic cyst. The drainage bag for the hepatic cyst infection was also inflated by positive pressure ventilation, suggesting a possible HBF. The ventilator was adjusted to minimize pressure on the fistula and prevent retrograde infection. The fistula eventually closed spontaneously. Appropriate antibiotic treatment and continuous drainage resulted in improvement of the hepatic cyst infection; however, the patient died due to respiratory complications. HBF should be suspected when positive pressure ventilation results in increased air at the hepatic infection site.

## INTRODUCTION

A hepatobronchial fistula (HBF) is a rare complication of several hepatic diseases; no previous case reports have described HBF associated with hepatic cyst infection [1]. An HBF is diagnosed by imaging and identification of clinical symptoms such as abdominal infection and respiratory failure [2]; it is important to minimize pressure on the fistula through drainage or other means [3]. In the case of ventilator management, positive pressure ventilation is required, making management even more difficult. We report the first case of hepatic cyst infection complicated by an HBF, diagnosed based on both characteristic computed tomography (CT) and clinical findings. Appropriate ventilator settings, as well as drainage management, eventually resulted in fistula closure.

## CASE REPORT

**Figure 1:**
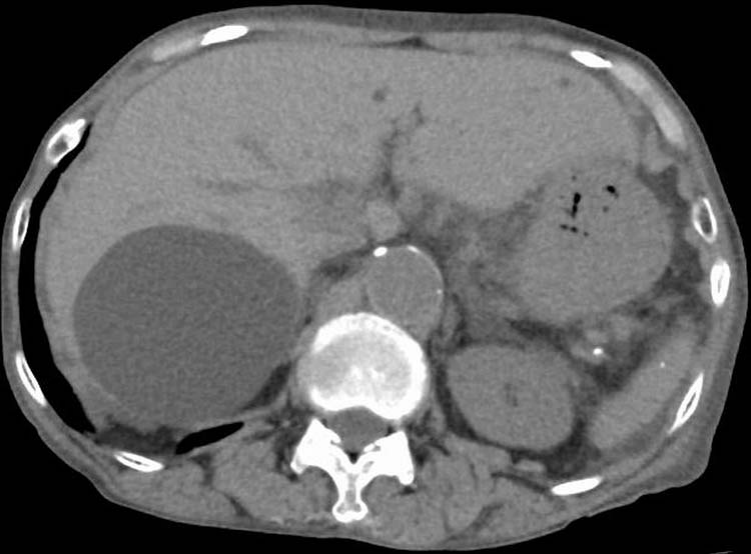
Abdominal CT 5 years prior to admission showing a cyst in the right hepatic lobe.

**Figure 2:**
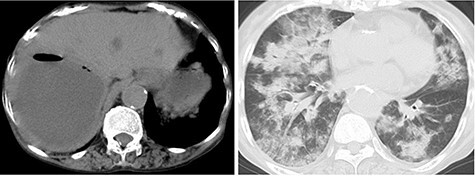
Abdominal and chest CT at admission showing a cyst containing air-fluid levels in the right hepatic lobe measuring 106 mm × 103 mm, and bilateral consolidation in the lungs.

An 81-year-old woman presented to the emergency department complaining of mild fever and right upper quadrant abdominal pain, that had lasted for one and a half months. She has been diagnosed with an asymptomatic hepatic cyst previously (Fig. 1). On examination, her body temperature was 36.1°C, blood pressure 116/77 mmHg, pulse 100/min and respiratory rate 25/min, with an O_2_ saturation of 86% on reserved mask at 10 L/min. Chest auscultation revealed bilateral crackles, and the abdomen was slightly distended. Laboratory examination results were as follows: white blood cell count, 16 100/μl; C-reactive protein, 14.52 mg/dl; AST, 24 U/L; ALT, 12 U/L; ALP, 465 U/L; γ-GTP, 91 U/L; T-Bil, 1.2 mg/dl and D-Bil, 0.4 mg/dl. Chest and abdominal CT showed bilateral consolidation in the lungs and a large cystic lesion with air in the right hepatic lobe (Fig. 2).

**Figure 3:**
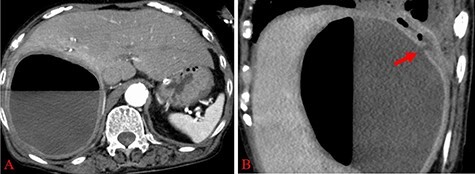
(**A**) Abdominal enhanced CT reveals a rapid increase of air within the hepatic cyst; (**B**) Abdominal enhanced CT showing disruption of the continuity of the wall of the hepatic cyst; the fluid component is continuing to the pleural cavity (red arrow).

**Figure 4:**
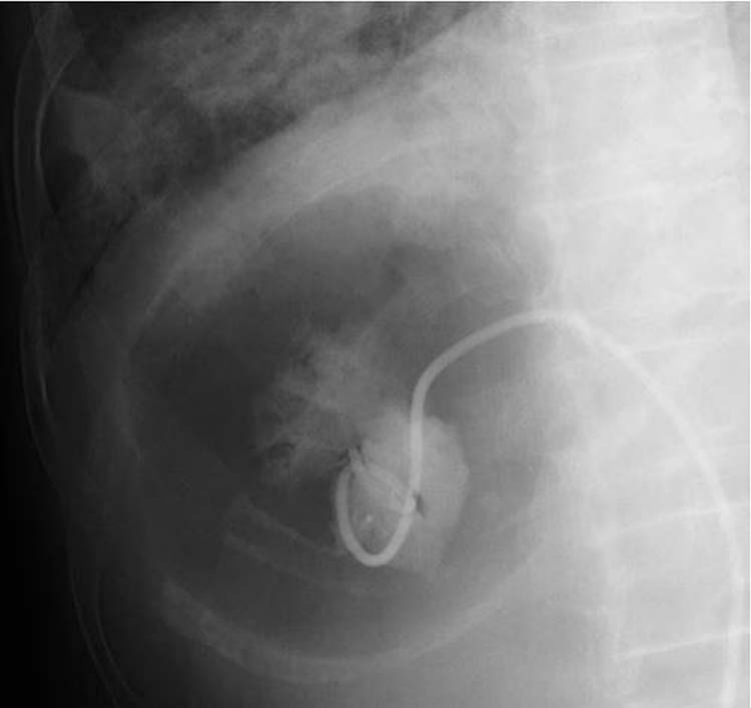
PTAD does not show any contrast agent leakage into the chest cavity.

She was intubated and treated with positive pressure ventilation, vasopressors and antibiotics (tazobactam/piperacillin and clindamycin). The PaO_2_/FiO_2_ ratio after intubation was 142 (positive end-expiratory pressure 7 cmH_2_O), and the patient was considered to have moderate acute respiratory distress syndrome (ARDS). Contrast-enhanced CT (8 h after the first) showed an increase in air in the hepatic cyst (Fig. 3A) and a potential connection between the hepatic infection site and peripheral bronchus (Fig. 3B). We performed percutaneous transhepatic abscess drainage (PTAD) and obtained 400 ml of creamy pus, and observed no leakage of contrast agent into the thoracic cavity (Fig. 4). In addition to CT findings, the drainage bag was inflated by positive pressure ventilation; this led us to believe that the patient had an HBF. The ventilation settings were adjusted to apply minimal pressure in order to prevent the drainage bag from inflating, while preventing retrograde infection with positive pressure ventilation.

**Figure 5:**
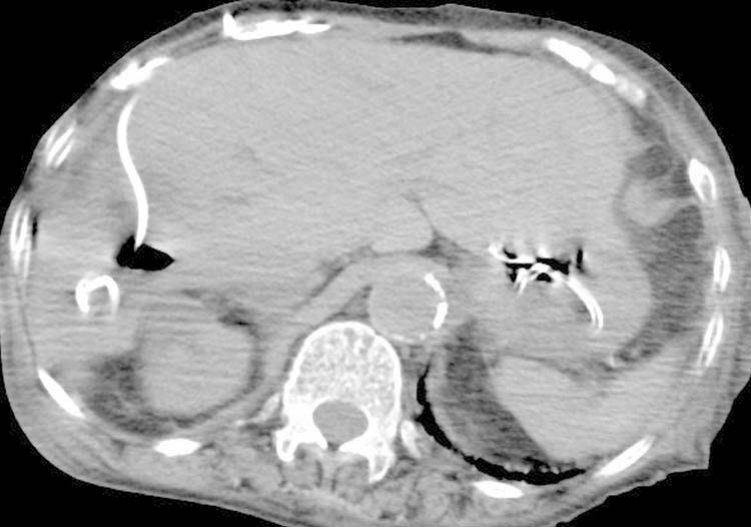
Abdominal CT shows a reduction in the size of the hepatic cyst infection.

**Figure 6:**
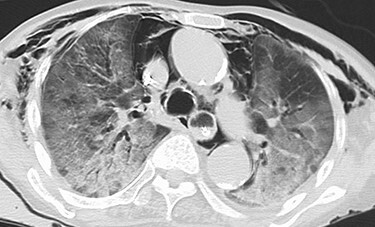
Chest CT showing mediastinal and subcutaneous emphysema.

Cultures of sputum and pus from the hepatic cyst were positive for *Escherichia coli*; this also supported the diagnosis of a HBF. We changed the antibiotics to cefazolin and metronidazole on Day 4; the fistula was considered to be clinically closed on Day 5, as the drainage bag was no longer inflated. Blood and sputum cultures were negative on Day 9. CT showed a reduction in the size of the hepatic lesion (Fig. 5), and her respiratory condition gradually improved. However, CT on Day 12 (Fig. 6) revealed mediastinal emphysema. Due to concerns regarding the possibility of exacerbation of the mediastinal emphysema with positive pressure ventilation and improvement of the PaO_2_/FiO_2_ ratio, she was extubated on Day 13. However, her respiratory condition worsened, and she was unable to withstand further invasive procedures. After discussion with her family, we halted invasive treatment on Day 16, and she died on Day 18.

## DISCUSSION

Hepatic cysts are asymptomatic and typically require no treatment. However, treatment is necessary when complications occur. Treatment strategies for infection include antibiotics, percutaneous drainage and surgical interventions. Initial independent antibiotic therapy fails in 70% of cases, eventually requiring percutaneous drainage or surgical treatment [4]. The mortality rate is relatively low [4]; however, complications due to HBF led to poor outcomes in our case. This case shows that patients with hepatic cysts are at risk of developing HBF due to infection.

There are five categories of HBF: congenital, hepatic hydatid disease or liver abscess, biliary tract obstruction secondary to tumors, injury and iatrogenic [1]. Few cases are caused by infection, which accounts for 10% or less [5]. A search of literature does not reveal any cases of hepatic cyst infection leading to HBF, although there are several case reports on HBF due to liver abscess, unrelated to hepatic cyst infection.

Endoscopic retrograde cholangiopancreatography (ERCP) and percutaneous transhepatic cholangiography (PTC) are useful for the detection of fistulae. CT and magnetic resonance imaging are also useful diagnostic tools, although they can fail to detect the fistulous tract itself [2]. In our case, we suspected HBF based on CT findings. A definite diagnosis was made based on the fact that the drainage bag inflated due to positive airway pressure, and the cultures of sputum and pus from the hepatic cyst were the same, although PTAD did not reveal the fistula. There are no case reports on drainage bag expansion due to positive pressure ventilation; this finding will be useful for future practice.

There is currently no standard treatment for HBF. Maintaining low pressure at the opening of the fistula is vital [3]. This can be accomplished by drainage, which may be accomplished by using a chest tube, ERCP, PTC or by surgical excision of the fistula tract. Clinicians prefer less invasive and non-surgical treatment, because the recovery rate is higher than that with traditional surgery [5]. However, in refractory cases, traditional surgery is necessary [6]. In our case, appropriate antibiotic treatment and drainage by PTAD were administered for the infected hepatic cyst; the patient responded well to the therapy. Ventilator settings were adjusted, and the fistula was considered to be clinically closed, because the drainage bag was no longer inflated by positive pressure ventilation. However, the patient had moderate ARDS at the time of initial treatment, and despite guideline-recommended respiratory management, she died due to progressive ARDS [7]. If the presence of the fistula itself is detrimental to the condition, early surgical intervention may be necessary; however, surgery was not performed in this case owing to the severity of the disease and the vulnerable health of the patient.
